# Tramadol/Paracetamol Fixed-Dose Combination for Chronic Pain Management in Family Practice: A Clinical Review

**DOI:** 10.5402/2013/638469

**Published:** 2013-04-11

**Authors:** Ignacio Morón Merchante, Joseph V. Pergolizzi, Mart van de Laar, Hans-Ulrich Mellinghoff, Srinivas Nalamachu, Joanne O'Brien, Serge Perrot, Robert B. Raffa

**Affiliations:** ^1^Centro de Salud Universitario Goya, c/O'Donnell 55, 28009 Madrid, Spain; ^2^Johns Hopkins University, Baltimore, MD 21287, USA; ^3^Association of Chronic Pain Patients, Houston, TX 77515, USA; ^4^Arthritis Center Twente (MST & UT), P.O. Box 50.000, 7500KA Enschede, The Netherlands; ^5^Department of Endocrinology, Diabetology and Osteology, Kantonsspital St. Gallen, 9007 St. Gallen, Switzerland; ^6^Kansas University Medical Center, Kansas City, KS 66160, USA; ^7^International Clinic Research, Overland Park, KS 66210, USA; ^8^Department of Pain Medicine, Beaumont Hospital, Beaumont, Dublin 9, Ireland; ^9^Service de Médecine Interne et Consultation de la Douleur, Hôpital Dieu, 75004 Paris, France; ^10^Department of Pharmaceutical Sciences, Temple University School of Pharmacy, Philadelphia, PA 19140, USA

## Abstract

The family practitioner plays an important role in the prevention, diagnosis, and early management of chronic pain. He/she is generally the first to be consulted, the one most familiar with the patients and their medical history, and is likely the first to be alerted in case of inadequate pain control or safety and tolerability issues. The family practitioner should therefore be at the center of the multidisciplinary team involved in a patient's pain management. The most frequent indications associated with chronic pain in family practice are of musculoskeletal origin, and the pain is often multimechanistic. Fixed-dose combination analgesics combine compounds with different mechanisms of action; their broader analgesic spectrum and potentially synergistic analgesic efficacy and improved benefit/risk ratio might thus be useful. A pain specialist meeting held in November 2010 agreed that the fixed-dose combination tramadol/paracetamol might be a useful pharmacological option for chronic pain management in family practice. The combination is effective in a variety of pain conditions with generally good tolerability. Particularly in elderly patients, it might be considered as an alternative to conventional analgesics such as NSAIDs, which should be used rarely with caution in this population.

## 1. Introduction

**Figure 1:**
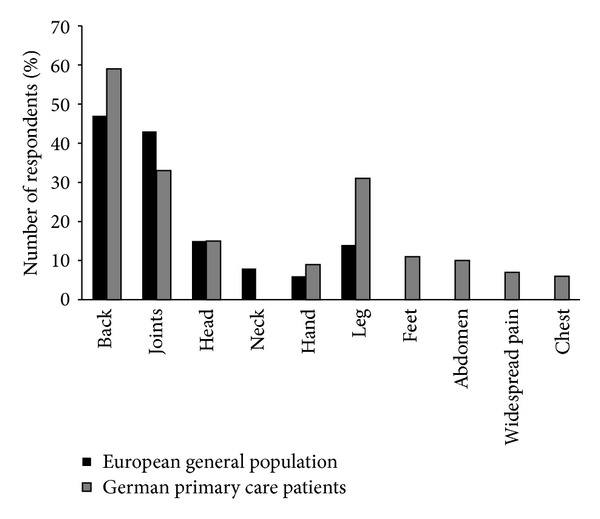
Main chronic pain locations reported for the general European population and in German primary care clinics (survey data) [1, 4].

**Figure 2:**
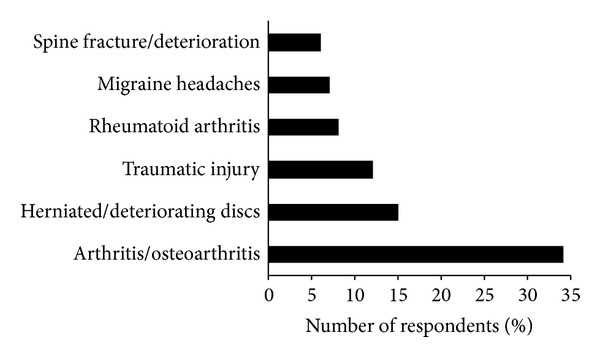
Main chronic pain indications in the general European population (survey data) [4].

One of the major reasons for patients presenting at a primary care practice is pain; according to surveys, around 40% of patients consult their family doctor mainly owing to pain, and 20–40% of those have been suffering from pain for more than 6 months [1, 2]. High prevalence rates for chronic pain have been reported in many countries [3–10]; for example, 20% of the European population are suffering from moderate-to-severe chronic pain (1-month prevalence, excluding cancer pain [3]). In the majority of cases (70%) the family practitioner is looking after these patients [4]. The most frequent indications associated with chronic pain in the family practice are of musculoskeletal origin, primarily affecting the back and joints (Figures 1 and 2) [1, 4]. 

Chronic pain affects quality of life and personal relationships and is often accompanied by depression, sleep disorders, and also low self-esteem [4]. In addition to the immense burden to the patient, it presents a substantial challenge and burden to society in terms of direct (healthcare resource utilization [11]) and indirect costs (e.g., social compensations, retirement pensions, loss of productivity, and skills [4, 12]). 

For many indications, a combination of nonpharmacological and pharmacological treatments will likely lead to the best outcome. Chronic pain management is basically a balancing act between the obvious goal of pain reduction and the goals of safety and quality of life of the affected patients. Whereas a number of medications are suitable for the short-term treatment of acute pain, patients with chronic pain require long-term, sometimes life-long treatment, and finding effective, safe, and tolerable pain treatments may become a challenge. In addition, many affected patients are elderly [7, 13, 14], which is not surprising as age-related degenerative diseases such as osteoarthritis are frequently associated with chronic pain. Thus, comorbidities and possible ensuing drug-drug interactions owing to polypharmacy and the adverse event profile of a given medication must all be taken into account in an individual patient's tailored treatment approach. 

A consensus meeting of pain specialists from different fields was held in November 2010 in Paris, France, in order to review and discuss the management of moderate-to-severe chronic pain with special emphasis on the use of paracetamol, NSAIDs, and tramadol/paracetamol as an example for fixed-dose combination analgesics. Discussions about available treatment options for chronic pain management and current guidelines and treatment recommendations for various conditions and patient populations were continued after the meeting; the final consensus on pain management options for pain indications frequently seen by a family practitioner is presented in this paper.

## 2. Pain Assessment 

Chronic pain can be categorized as nociceptive pain (caused by tissue damage or disease), neuropathic pain (caused by damage or disease of the peripheral or central nervous system), or mixed pain with both nociceptive and neuropathic components [15]. In addition, the location of pain may differ from the site of origin of pain, as is the case with referred pain and radicular pain. Successful pain management initially requires a thorough diagnostic work-up, through patient history, physical examination, and diagnostic tests in order to identify

- the location and cause of pain;
- the frequency with which the pain occurs and potential exacerbating and relieving factors;
- previous treatment received by patient, and its efficacy;
- the severity of pain, and its quality (e.g., dull, aching, stabbing, lancinating, burning, etc.);
- the chronification stage of pain;
- the impact of pain on the patient (e.g., quality of life, loss of productivity), his/her family or caregivers.

Initial pain assessment should also include documentation of pain frequency (continuous, intermittent, or continuous with intermittent flares), of presence or absence of triggers, and of presence or absence of somatosensory abnormalities such as increased or decreased perception of stimuli (e.g., hypo- or hyperalgesia, allodynia, or numbness). 

**Figure 3:**
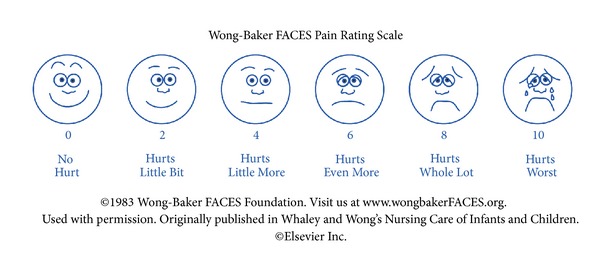
Wong-Baker FACES pain rating scale. This is a self-assessment tool. Patients need to be able to identify which face most accurately depicts the pain they are experiencing.

Simple scores should be used to assess pain intensity in order to monitor the progression/improvement of pain with a time-saving method during the family practitioner visit. Pain intensity is usually assessed using 11-point rating scales with 0 indicating “no pain” and 10 indicating “worst imaginable pain” or along a 100 mm horizontal line, with ratings measured from the left edge (Table 1). Visual analogue (VAS) and numerical (NRS [16]) and verbal (VRS) rating scales are available both for the assessment at the family practice and for patients' self-assessments. In addition, Wong-Baker FACES scales (Figure 3 [17]) are used for children and adults who are unable to communicate their pain such as patients with cognitive impairment. Pain intensity assessments are widely used as efficacy parameters for a given medication, both in clinical trials and in clinical practice. 

For a comprehensive diagnosis of chronic pain, a determination of pain type is also necessary and should include the use of neuropathic pain assessment tools such as pain DETECT [18], DN4 (Douleur Neuropathique en 4 questions [19], Table 2), or LANSS (Leeds Assessment of Neuropathic Symptoms and Signs [20]), which have been validated in several languages. The assessment of neuropathic pain in primary care has recently been extensively reviewed [21].

One way of defining pain as chronic has been on the basis of its duration, that is, if it lasts for more than 3 or 6 months [22, 23]; however, a standard, internationally accepted definition for chronic pain is not available. In Germany, chronicity staging with the validated Mainz Pain Staging System [24] is part of the German Pain Questionnaire, a standardized and validated tool for chronic pain therapy [25]. The three stages—I (at risk of chronification), II (chronification), and III (marked chronification)—indicate the progression of the disease. Currently, attempts are underway to include psychological and behavioral factors in a definition of chronic pain, with the objective of including all aspects of a patient's pain in an integrated pain management strategy [26]. The assessment of chronicity is important in order to assess the risk of future chronic pain in a given patient and to rapidly initiate adequate pain management [26]. 

Initial and follow-up pain intensity assessments are essential but not sole indicators for successful or unsuccessful treatment. Other factors such as improved mobility or independence of the patient, working ability, improved coping and psychological status, and overall quality of life might be equally important to the patient (see the following).

## 3. Pain-Related Comorbidities and Quality of Life

Chronic pain has an enormous impact on quality of life and daily activities of the patient. It affects sleep in the majority of patients, impairs physical activities, the ability to attend to household chores, and employed work, and restricts many patients in terms of relationships and social activities [27]. Additional burden arises for the patient through pain-related comorbidities such as depression and anxiety. According to a recent survey among chronic pain patients with moderate-to-severe pain (defined as pain ≥5 on an 11-point scale for six months or more) in the European Union, 21% had been diagnosed with pain-related depression [4]. Whereas pain treatment in the past primarily targeted the reduction of pain intensity, maintaining or improving quality of life of the patients is increasingly being recognized as an equally important goal. Thus, in addition to the diagnosis of pain, quality of life parameters and pain-related comorbidities ought to be assessed and taken into consideration in treatment decisions. Some examples for commonly used assessment tools are given in Table 1; many international tools have been translated and validated in various languages. In addition to overall quality of life questionnaires such as the SF-36-Health Survey [28], scales determining depression, anxiety, and stress [29, 30], and disease-specific tools such as the Western Ontario and McMaster Universities (WOMAC) Osteoarthritis Index [31, 32] have been developed. Recently, the CHANGE PAIN scale was introduced, a simple tool easy to use in primary practice, which can initially be used to record pain intensity and define individual treatment goals but can subsequently determine patient-centered perceptions of change [33]. 

Given primary practice reality with a short-time frame available for each individual patient, many physicians may shy away from the extra time spent in completing and evaluating additional questionnaires. However, these tools may enable a more efficient and thus in the long-term time-saving approach. In particular, focusing on the patient's individual needs may reduce a substantial burden for the patient and avoid long periods of unnecessary suffering owing to unsuitable treatment approaches. 

## 4. Pain Management by Family Practitioners

The family practitioner plays an important role in the prevention, diagnosis, and early management of chronic pain. He/she is usually the physician who is most familiar with the patient and his/her medical history, keeps an overview on the various therapies prescribed by him-/herself and other specialists, and is likely to be the first to be alerted in the case of inadequate pain control or if safety issues owing to comorbidities or comedications arise. Since the family practitioner usually also builds a trusting relationship with the patient, the patient is most likely to disclose concerns, potential side effects of medications, effects of the treatments/pain on his/her daily life and social network, and so forth. The family practitioner might be able to identify unmet needs of the patient, requirements for patient information, and education and potential requirements to involve the family/partners in the overall treatment plan and care. For many indications, in particular musculoskeletal diseases, a multimodal treatment approach combining both nonpharmacological and pharmacological treatments will likely lead to the best outcome in terms of pain relief and improvement of quality of life. The family practitioner should be at the core of a multidisciplinary team for pain management including, for example, pain specialists, physiotherapists, and others. Nonpharmacological treatments may include changes in diet, weight loss, changes in lifestyle, exercise, or massage that can be easily accessible to primary care physicians. Other options like acupuncture, thermotherapy, transcutaneous electrical nerve stimulation (TENS), surgery, and many others are beyond the scope of this paper. This section will focus on general considerations concerning available pharmacological treatment options.

### 4.1. Paracetamol

Paracetamol (also known as acetaminophen or APAP) is a very popular medication, which is available over-the-counter as well as in prescription medications. It has analgesic and antipyretic but, unlike nonsteroidal anti-inflammatory drugs, no confirmed anti-inflammatory properties, although there have been studies pointing to some anti-inflammatory effects [38, 39]. Its exact mode of action is still poorly understood and several mechanisms have been proposed, which seem to point to a largely central effect [40]. In healthy individuals, a daily maximum dose of 4 g is considered relatively safe [41], given a considerable interindividual variability. However, certain conditions such as chronic alcohol abuse, malnutrition, existing liver disease, and concurrent use of drugs, which induce cytochrome P450 enzymes, can lead to hepatotoxicity even at therapeutic dosages [42]. In Europe and North America, a large proportion of cases of acute liver failure are due to paracetamol [43]. Paracetamol overdoses are often unintentional [42] and likely occur because many patients are unaware of the fact that their “harmless” cold remedies or headache tablets can also contain paracetamol. Patients therefore need to be properly educated if paracetamol-containing medications are prescribed. 

### 4.2. NSAIDs

NSAIDs are very effective drugs with antipyretic, anti-inflammatory, and analgesic efficacy rendering them useful for many acute pain conditions. They are widely used over-the-counter and prescription products and are contained in many combination formulations. 

NSAIDs are associated with gastrointestinal, cardiovascular, and renal side effects and mortality with an estimated 3500 to 16,500 deaths per year due to NSAID-related gastrointestinal bleeding in USA [44]. In addition, NSAIDs also interact with many other medicinal products [45–48], and special caution and close monitoring are recommended for patients with conditions such as gastrointestinal disorders, renal, cardiac, or hepatic impairment, and hypertension and patients with a history of asthma and seasonal allergic rhinitis (since they act as bronchoconstrictors). Following the discovery of cyclooxygenase (COX)-2 and in an attempt to overcome the gastrointestinal side effects of NSAIDS, COX-2-selective NSAIDs were developed. Rofecoxib was withdrawn from the market approx. 5 years later because of cardiovascular risks, shortly followed by warnings and dose restrictions for all NSAIDs. 

Owing to these safety issues, all NSAIDs should be used for the shortest possible duration at the lowest effective dose. In the elderly in particular, NSAIDs should be used “rarely and with extreme caution in highly selected individuals” [49]. In general, NSAIDs are not suitable for long-term treatment of chronic pain conditions.

### 4.3. Opioids

Opium has been used since antiquity for treating pain. In modern times, synthetic opioids have been added to the naturally derived compounds. Some of the side effects and special issues related to opioids might restrict their long-term use, at least in some patients [50]. Constipation is a frequent problem requiring comedication for most strong opioids [51]. In addition, respiratory depression is likely to cause problems in special patient groups, such as asthmatics and cardiovascular patients with impaired lung function. 

Opioids are associated with different levels of tolerance, dependence, and addiction potential. These are three distinct phenomena. Tolerance describes the state in which a patient requires increasingly higher doses of the opioid to achieve the same level of pain relief. In some cases, tolerance is a normal response to opioid therapy; it can sometimes be managed by opioid rotation, that is, switching to other opioids [52]. Manifestations of opioid-induced hyperalgesia mimic tolerance [53]. A patient with opioid-induced hyperalgesia should, however, be tapered off opioids, whereas a patient with tolerance may require a judicious and supervised increase of the opioid dose [53]. Dependence occurs when discontinuation of the substance results in withdrawal symptoms; dependence is a normal and expected result of long-term opioid therapy and is typically managed by tapering the drug rather than abruptly stopping it [54]. Addiction has been defined as the continuing use of a substance (or behaviour) despite adverse consequences to the user [55]. Opioid-addicted patients are dependent, but not all opioid-dependent patients are addicted. A newer term in use is “inappropriate use of opioid,” a continuum of behaviors involving the intentional or inadvertent misuse of opioids, particularly prescribed opioids [56]. A variety of opioid analgesic products have been designed in abuse-deterrent or abuse-resistant formulations, aimed at making it more difficult to tamper with the products [57, 58].

However, these dangers are frequently overestimated, and opioids are often the only choice for severe pain and can be a good choice for special patient groups such as the elderly. They also present an alternative when long-term NSAID treatment is not recommended. The transdermal formulations of fentanyl and buprenorphine in particular appear to be effective with low toxicity and good tolerability profiles, especially at low doses [59]. 

### 4.4. Tramadol

Tramadol has been on the market since 1977 and is considered a type of opioid. However, it is different from most other opioids because of its multiple mechanism of analgesic action (binding to *μ*-opioid receptors and inhibition of neuronal reuptake of norepinephrine and serotonin) [60, 61]. Tramadol has been shown to be effective in different acute and chronic pain states [62, 63]; its extended-release formulations are thought to be particularly suitable for long-term treatment of multimechanistic pain conditions such as osteoarthritis, low back pain, and neuropathic pain [64]. Tramadol has no known anti-inflammatory effects. 

Side effects include nausea, vomiting, dizziness, drowsiness, sweating, and dry mouth; reports of drug dependence and abuse are rare [65]. Isolated cases of serotonin syndrome have been reported during concomitant use of tramadol in combination with other serotoninergic medicinal products such as selective serotonin reuptake inhibitors (SSRIs) or with MAO inhibitors [65]. Unlike other opioids, tramadol has no clinically relevant effects on respiratory or cardiovascular parameters at recommended doses [66]. For chronic pain, 50 mg or 100 mg tramadol is administered every 4 to 6 h, with a total daily dose not in excess of 400 mg; the dosage interval should be extended in elderly patients and patients with renal or hepatic impairment [65]. 

### 4.5. Topical Analgesics

Topical analgesics are an important pain management option which provides localized pain relief. Overall, this group is efficacious with a good safety profile [67]. Due to reduced or no systemic action, the risk of systemic side effects is markedly reduced which might increase patient compliance, safety, and quality of life. Typical side effects include skin irritations which are often mild. Most topical analgesics are available as patches, ointments, or creams [67], many as over-the-counter products. Examples are the 5% lidocaine medicated plaster [68], topical NSAIDs [69], and the 8% capsaicin patch [70]. 

### 4.6. Anticonvulsants

The clearest evidence for the efficacy of anticonvulsants in pain relief was found in patients suffering from neuropathic pain [71]. Several mechanisms of action have been reported for anticonvulsants, among them the blockage of voltage-gated calcium channels (gabapentin, pregabalin, etc.) and the blockage of voltage-gated sodium channels (carbamazepine, oxcarbazepine, lamotrigine, lacosamide, etc.) [72]. 

Dose-related side effects occur with all anticonvulsants; the majority are CNS related (i.e., somnolence, dizziness, ataxia, and headache) [72]. There are also specific, sometimes significant adverse reactions depending on the prescribed anticonvulsant such as hyponatremia, leukopenia, thrombocytopenia and hepatotoxicity for carbamazepine and weight gain, and blurred vision and edema for gabapentin and pregabalin [71].

### 4.7. Antidepressants

There is evidence of antidepressant analgesia independent of their effect on depression or other psychiatric disorders [73]. Tricyclic antidepressants (TCAs) provide pain relief in a variety of conditions but are associated with side effects such as sedation, dizziness, blurred vision, constipation, and dry mouth, which can be treatment limiting. Cardiac toxicity is a concern, and it is recommended to prescribe TCAs with caution in patients with ischemic cardiac disease or ventricular conduction abnormalities [74]. Amitriptyline and other tertiary TCAs are listed on the recently revised Beers list of potentially inappropriate medication use in older adults with a strong recommendation to avoid their use because they are highly anticholinergic, sedating and cause orthostatic hypotension [75].

The selective serotonin norepinephrine reuptake inhibitors (SNRIs), duloxetine and venlafaxine, are first-line treatments for neuropathic pain, and duloxetine and milnacipran are recommended for fibromyalgia treatment. Overall, they are better tolerated than TCAs with nausea as the main side-effect. Caution is advised for venlafaxine in patients with cardiac disease [74]. 

### 4.8. Glucocorticoids

Glucocorticoids have clinically important anti-inflammatory and immunosuppressive effects which are, among others, used in the treatment of rheumatic diseases [76]. Safety concerns for higher doses or long-term treatment include amongst others glucocorticoids-induced osteoporosis and risk of fracture [77], immunosuppression [78], increased risk of infections, weight gain, leg edema, thinning skin, Cushing's syndrome, hypertension, glaucoma, cataracts, shortness of breath, and sleep disturbances [79], and the onset or worsening of diabetes [80]. Patients with established rheumatoid arthritis should be made aware of long-term complications with glucocorticoids and all other treatment options should be offered before glucocorticoid treatment is maintained in the long-term [81].

### 4.9. Combination Products

Since most pain conditions involve more than one underlying pain generating process and pain is transmitted via a large number of different pathways, a practical treatment approach is using drugs or drug combinations with different mechanisms of action and thus different targets. However, not all combinations are ideal because a combination of two or more individual compounds may lead to additive or even synergistic analgesic effects but might also lead to a higher than anticipated side-effect rate. Thorough testing of combinations and determining the right dose ratio of the individual components are therefore required. A recent review covers these issues in detail [61]. 

Fixed-dose combination analgesic products reduce the pill burden and may require lower dosages than the individual compounds. They are, however, inflexible, and doses may not be ideal for particular patients. Many fixed-dose combination analgesics contain paracetamol, and patients may exceed the recommended daily paracetamol intake of 4 g for healthy adults [41]. Family practitioners need to educate their patients on this issue.

Fixed-dose combinations with an opioid include codeine/paracetamol, oxycodone/paracetamol, tramadol/paracetamol, and others. These products may be opioid sparing, because they provide effective analgesia at lower opioid doses than opioids taken in monotherapy. This was shown in two studies comparing tramadol/paracetamol with tramadol monotherapy in the management of subacute low back pain [82] and pain following ambulatory hand surgery with iv regional anesthesia [83]. Fixed-dose combination treatment reduced tramadol consumption by 24% in both studies and resulted in significantly fewer side effects than with tramadol monotherapy.

Analgesic synergy of fixed-dose combination products for pain relief in humans has, to our knowledge, so far only been demonstrated for tramadol/paracetamol [84]. Details concerning the experience with this combination are given below for individual indications. 

## 5. Pain Indications Frequently Encountered by the Family Practitioner

This section lists the most common chronic pain indications encountered by the family practitioner and available treatment guidelines/recommendations for the individual indications. A summary of the studies investigating treatment with tramadol/paracetamol for a given indication is included.  Table 3 summarizes the design of the studies mentioned below.

### 5.1. Musculoskeletal Pain

The majority of chronic pain patients in primary care suffer from musculoskeletal pain [1]. Indications reviewed separately below are osteoarthritis, low back pain, and rheumatoid arthritis. Three studies assessed tramadol/paracetamol for musculoskeletal pain of different origins and are discussed here [85–87]. The two latter studies are of particular interest to the family physician because they observed pain management in the clinical practice setting.

The efficacy and tolerability of tramadol/paracetamol were compared to codeine/paracetamol in adult patients with chronic nonmalignant low back pain, osteoarthritis pain, or both [85]. At baseline, three-quarters of patients in both treatment groups presented with at least moderate pain. Pain relief was comparable between treatments and associated with a similar incidence of overall adverse events except for somnolence (24% versus 17%; *P* = 0.05) and constipation (21% versus 11%; *P* < 0.01), which was more common for codeine/paracetamol treatment. Patients completing this study could participate in an 23-month open-label extension study [88]. They received a mean daily tramadol/paracetamol dose of 157 mg/1363 mg. Pain relief was maintained during the extension period. Overall treatment efficacy was rated very good or excellent by 39% of the patients and 40% of the physicians. Twenty-four percent of the patients discontinued prematurely due to adverse events.

The ELZA study assessed efficacy and safety of tramadol/paracetamol in 5495 patients aged over 12 years who presented with moderate-to-severe pain (for more than 3 months in 15.6% of the patients) to general practitioners in France [86]. The most common origin of pain was musculoskeletal (24.9% osteoarthritis, 20.1% low back pain, and 13.9% spinal nerve root compression). Treatment reduced the mean pain intensity at baseline from 6.3 ± 1.7 (11-point NRS scale) to 2.3 ± 1.9 at final assessment (*P* < 0.001). Quality of sleep improved in 54.9% of all patients. A total of 4.2% of the patients reported adverse events, most commonly gastrointestinal disorders.

The SALZA study assessed the clinical benefits of tramadol/paracetamol in 2663 patients aged ≥65 years with moderate-to-severe pain in French general practices [87]. The most common origin of pain was musculoskeletal (59.1% arthrosis, 33% low back pain, 16.9% spinal nerve root compression, and 15.8% other rheumatological pathologies). The mean pain intensity at baseline of 6.1 ± 1.6 (11-point NRS scale) was reduced by 3.1 points; 64.8% experienced complete pain relief, and the majority (90.5%) were satisfied with the treatment. Discontinuation of treatment due to insufficient efficacy or adverse events occurred in 1.5% and 2.5% of all patients, respectively. Adverse events were reported by 4.5% of the patients, mainly gastrointestinal disorders (4.1%).

These studies show the efficacy of fixed-dose tramadol/paracetamol with a good safety profile in the treatment of chronic musculoskeletal pain both under randomized study conditions and, more importantly, in clinical practice. The clinical benefits in the elderly in particular are noteworthy and suggest tramadol/paracetamol as an alternative to conventional analgesics such as NSAIDs, which should be considered rarely with extreme caution in this population [49]. 

#### 5.1.1. Osteoarthritis

Prevalence of osteoarthritis (OA) increases with advancing age; the greatest disease burden is attributable to the involvement of the hip or knee joints [89]. Pain is the overriding clinical issue [90]. Complex underlying pain mechanisms [91] might require the administration of pain medications from different medication classes to achieve sufficient analgesia.

OA management is focused on symptom control, prevention of disease progression, minimization of disability, and improvement of quality of life [92] and requires a combination of nonpharmacological and pharmacological measures. Recommendations for initial analgesic treatment include paracetamol and topical NSAIDs (for hand and knee) ahead of oral NSAIDs including COX-2 inhibitors [93–95]. The latter should be used at the lowest effective dose, and long-term use should be avoided. Weak opioids and topical capsaicin and intra-articular corticosteroid injections as an adjunct can also be considered. However, stronger opioids should only be used for severe pain in exceptional circumstances [93] or in symptomatic knee or hip OA following insufficient response to both nonpharmacological and pharmacological treatments and where patients are not suitable for total joint arthroplasty [94]. 

OA pain management using tramadol/paracetamol fixed-dose combination as an add-on treatment to NSAIDs or COX-2 inhibitors or as monotherapy was investigated in four studies [96–99]. Adding the fixed-dose combination to the treatment regimen of patients with OA flare pain (hip or knee) significantly reduced mean baseline pain intensity scores from 2.4 ± 0.5 to 1.3 at the final visit compared to placebo (*P* < 0.001) on a 4-point scale (from 0 = none to 3 = severe) [96]. Treatment-related adverse events occurred in 24.4% patients receiving tramadol/paracetamol and in 8.1% of placebo patients; withdrawal due to adverse events was reported for 12.7% patients on fixed-dose combination and 5.4% of placebo patients. Tramadol/paracetamol also provided significantly superior pain relief compared to placebo (*P* = 0.019) in the subgroup of 113 elderly patients [100]. 

Patients with knee or hip OA and with at least moderate pain (>69 mm in a 100 mm VAS scale) despite treatment with stable doses of celecoxib or rofecoxib received tramadol/paracetamol or placebo as add-on therapy for 3 months [97]. Pain intensity scores following combination treatment were significantly lower than those for placebo (41.5 ± 26 versus 48.3 ± 26.6, *P* = 0.025). Improvement in WOMAC physical function was also significantly better than that in the placebo group (*P* = 0.049). Withdrawal due to adverse events occurred in 13.1% tramadol/paracetamol and 3.9% placebo patients. No serious adverse events occurred that were related to the study medication.

Korean patients with knee OA and moderate pain despite stable doses of meloxicam or aceclofenac received add-on treatment with tramadol/paracetamol for 4 weeks [98]. All patients with reduced pain (<4 on the NRS) were then randomized to monotherapy of either tramadol/paracetamol or NSAID treatment for a further 8 weeks. The 97 patients with significant pain reduction and significant improvements of the WOMAC OA index score (both *P* < 0.0005) following the 4-week add-on therapy maintained their pain-improved state with either agent for the next 8 weeks. One tramadol/paracetamol patient withdrew due to adverse events. 

A second Korean study investigated the effect of tramadol/paracetamol titration on the development of adverse events in patients with knee OA on stable NSAID therapy [99]. Patients were randomized to either one 37.5 mg tramadol/325 mg paracetamol tablet tid for 2 weeks or titration to this dose over 7 days. The discontinuation rate due to adverse events was significantly lower in the titration group (10.5% versus 26.2% for nontitration, *P* < 0.001). 

In all studies, the most common treatment-related adverse events were nausea, vomiting, constipation, dizziness, and somnolence. These adverse effects can be avoided in the majority of patients with a thorough titration of tramadol/paracetamol.

These studies suggest the effectiveness of tramadol/paracetamol as an add-on option to NSAIDs in the management of OA pain. However, in light of today's knowledge about NSAIDs, long-term treatment with NSAIDs cannot be considered safe any longer and maintenance treatment with tramadol/paracetamol with NSAIDs for flares is likely the safer option.

#### 5.1.2. Low Back Pain

Low back pain is very common and is nonspecific for the majority of patients [101]. It can arise from many different causes and can comprise both nociceptive and neuropathic mechanisms [102]. A multimodal and individualized treatment approach is therefore required [103]. Both the ACPC/APS and NICE guidelines recommend physical activity and exercise, manual therapy (including spine manipulation), acupuncture, and cognitive-behavioral therapy as nonpharmacological options [104, 105]. According to the NICE guidelines, pharmacological treatment should start with paracetamol followed by NSAIDs (combined with a proton pump inhibitor in persons >45 years) and/or weak opioids (which seem to include fixed-dose combinations) [105]. TCAs can be offered if these medications are insufficient but SSRIs are not recommended. Strong opioids can be given in the short-term for severe pain. Paracetamol and NSAIDs provide pain relief for nociceptive pain but are largely ineffective in neuropathic pain [21]. A combination of treatments is therefore often required to manage both components of chronic low back pain [103].

The efficacy of tramadol/paracetamol for the treatment of chronic low back pain was assessed in two studies [106, 107]. Both were double-blind, randomized studies in patients with at least moderate chronic lower back pain receiving either fixed-dose combination treatment or placebo over a 3-month period. 

In the first study, patients had a mean baseline pain score (100 mm VAS pain scale) of 71.1 mm (tramadol/paracetamol group) and 68.8 mm (placebo group) [106]. Combination treatment significantly improved pain scores (to 44.4 mm versus 52.3 mm for placebo, *P* = 0.015) compared to placebo and provided better pain relief (*P* < 0.001) and improved scores on measures of functioning and quality of life. Cumulative incidence of discontinuation due to insufficient pain relief was significantly lower for the combination product (22.1% versus 41% for placebo, *P* < 0.001). Treatment-related adverse events were reported for 23.6% of tramadol/paracetamol and 3.8% placebo patients with discontinuation rates due to adverse events more frequently observed for the active agent (18.6% versus 5.7%). 

The mean baseline pain score of the patients included in the second study was 68 mm [107]. Tramadol/paracetamol treatment reduced pain more effectively than placebo (final pain scores 47 mm versus 63 mm, *P* < 0.001) and improved quality of life and emotional and mental health. The withdrawal rate due to adverse events was 28.1% for tramadol/paracetamol and 7.6% for placebo. Main treatment-related adverse events in the combination group were nausea (12%), dizziness (10.8%), and constipation (10.2%). 

In summary, the two studies show moderate benefits of tramadol/paracetamol treatment compared to placebo for patients with chronic low back pain over a 3-month period. However, a multimodal approach is often required to achieve adequate pain relief. 

#### 5.1.3. Rheumatoid Arthritis

Rheumatoid arthritis (RA) is a common inflammatory disease characterized by persistent synovitis, systemic inflammation, and autoantibodies [111]. The etiology is multifactorial; prevalence rises with age and is highest in elderly women [111]. Besides the destruction of cartilage and underlying bone, RA often leads to cardiovascular and other comorbidities. Disease-modifying antirheumatic drugs are the mainstay of RA treatment but analgesics are commonly required from the very beginning to control pain. NSAIDs are recommended for pain management in early arthritis after careful evaluation of gastrointestinal, renal, and cardiovascular status [112] and in a stepped approach coprescribed with a proton pump inhibitor in the short term [113] but are not appropriate for long-term disease control [81, 112, 113]. Analgesics such as paracetamol, codeine, or fixed-dose combinations are recommended to potentially reduce the need for long-term NSAID or COX-2 inhibitor treatment [81].

The administration of tramadol/paracetamol in RA pain was investigated in one study. Patients with RA pain inadequately controlled by NSAIDs and disease-modifying antirheumatic drugs received one tramadol/paracetamol tablet tid or matching placebo for one week as add-on to their treatment regimen [108]. Mean pain intensity (100 mm VAS scale) at baseline was 60.2 ± 14.5 mm for the combination and 61.3 ± 17.6 mm for placebo and improved significantly more in the tramadol/paracetamol group (47.2 ± 20 mm versus 53.8 ± 16.6 mm for placebo; *P* = 0.018). Mean daily pain relief scores were also significantly greater (*P* = 0.037) but physical function did not differ between the groups. Adverse events (57.6% versus 22.4% for placebo) and discontinuations due to adverse events (19% versus 3%) were significantly more common in the active group (*P* < 0.001). This study indicates some short-term efficacy of tramadol/paracetamol in RA pain management. In light of the long-term safety problems with NSAIDs, alternative long-term maintenance treatment with tramadol/paracetamol accompanied by add-on NSAIDs treatment for flares should be investigated in further studies. 

### 5.2. Painful Diabetic Polyneuropathy (DPN)

Diabetic polyneuropathy is a frequent long-term complication of diabetes. In approx. 6% of type 1 and 18% of type 2 diabetic patients, this condition is associated with neuropathic pain which has a profound impact on quality of life [114]. Traditional analgesics such as paracetamol and NSAIDs are largely ineffective in all types of neuropathic pain [21]. There are a number of guidelines available, and the ones published in the last two years were recently summarized and compared [115]. According to this paper, all guidelines recommend TCAs and duloxetine as first-line agents except for the American Academy of Neurology (AAN) guidelines which recommend only pregabalin [116]. Additional first-line medications according to the European Federation of Neurological Societies (EFNS) guidelines are gabapentin, pregabalin, and venlafaxine [117]. 

Two studies have assessed the efficacy of tramadol/paracetamol for painful DPN [109, 110]. In the first study, diabetic patients (94% type 2) with daily painful DPN in the lower extremities for the past 3 months received tramadol/paracetamol or placebo for 66 days [109]. Patients presented with average daily pain scores of 7.1 ± 1.4 and 7.1 ± 1.3, respectively (11-point NRS scale). Supplemental paracetamol use was permitted during the treatment phase but could not exceed 4 g daily from all sources. Treatment with tramadol/paracetamol reduced the average daily pain more effectively than placebo (−2.71 versus −1.83, *P* = 0.001) with a greater number of treatment responders (≥50% pain reduction: 37.5% versus 21.9%, *P* = 0.003). Significantly greater improvements than placebo were also observed for further pain measures, sleep interference, global impression, and several quality of life measures and mood. The adverse event rate was similar for both groups with nausea (11.9% versus 3.3%), dizziness, and somnolence (both 6.3% versus 1.3%) more common under combination treatment. 

The second study compared the efficacy of tramadol/paracetamol and gabapentin in type 2 diabetes with painful symmetric neuropathy in the lower limbs [110]. Mean pain intensity was reduced with no significant difference between treatments (*P* = 0.744). Improvements in further pain measures, sleep interference, and quality of life were comparable between groups. The incidence of adverse events was not significantly different between treatments except for nausea/vomiting (8.9% tramadol/paracetamol, 1.2% gabapentin, *P* = 0.03), and similar withdrawal rates due to adverse events were reported (13.9% versus 13.1%).

Overall, the two studies suggest that fixed-dose tramadol/paracetamol is well tolerated and leads to pronounced pain relief in the treatment of painful DPN. 

### 5.3. Elderly Patients

Pain is a common occurrence in elderly patients living in the community. Most elderly patients (85%) experience moderate or severe pain; for a third of them this pain is continuous [14]. Musculoskeletal pain is the predominant type of pain. Pain is often underrecognized and undertreated with significant consequences for mobility, mood, restrictions in daily activities, and social activities which can all greatly affect quality of life. Pain management is challenging. Elderly patients often suffer from comorbidities and require various medications, which could increase the risk of drug-drug interactions and limit the range of drugs appropriate for treatment. Pharmacokinetic and metabolic changes with advancing age resulting in different drug sensitivities compared to younger patients also need to be considered when prescribing medication. The recently updated Beers criteria for potentially inappropriate medication use in older adults provide the family practitioner with information and recommendations for different medication classes including pain medication [75]. Caution is advised when prescribing NSAIDs; the American Geriatrics Society recommends to consider NSAIDs “rarely, with extreme caution, in highly selected individuals” and to avoid their use in patients ≥75 years of age [49]. 

The efficacy and safety of tramadol/paracetamol fixed-dose combination were assessed in a subset of patients ≥65 years of age with OA flare pain in a placebo-controlled, randomized study [100] and in elderly patients with predominantly musculoskeletal pain participating in the larger-scale noninterventional, observational SALZA study [87]. Both studies were described previously; both showed that tramadol/paracetamol was efficacious with a good safety profile for the elderly participants. Older patients (≥75 years old) in the observational ELZA study also showed significantly reduced pain intensity following treatment with the fixed-dose combination; 65.1% reported important or complete pain relief [86]. For patients aged over 75 years, the minimum interval between doses should be not less than 6 hours, due to the presence of tramadol [118].

## 6. Conclusions

- Pain is one of the major reasons for patients to consult their family practitioner.
- The family practitioner plays an important role in the prevention, diagnosis, and early management of chronic pain and should be at the core of the multidisciplinary team for pain management.
- The diagnostic work-up of pain should include quality of life parameters and an evaluation of the specific situation of the patient.
- The goal of pain management is not only pain relief, but also should include improvements in restrictions of daily activities and social life and in quality of life to increase the overall well-being of the patient.
- Long-term treatment of chronic pain is often associated with safety and tolerability issues and must be individualized for each patient.
- NSAIDs are useful short-term analgesics but are associated with gastrointestinal, cardiovascular, and renal side effects and mortality. Their long-term use in the management of chronic pain needs to be carefully weighted against potential side effects.
- Pain is often multimechanistic. Fixed-dose combination analgesics combine compounds with different mechanisms of action and might thereby be useful in the treatment of multimechanistic pain. They might provide a broader analgesic spectrum, potentially synergistic analgesic efficacy, and an improved benefit/risk ratio.
- Analgesic synergy was demonstrated for the fixed-dose combination tramadol/paracetamol. The combination was effective in a variety of different pain conditions frequently encountered by the family practitioner and showed good tolerability.
- Tramadol/paracetamol might be considered in particular in elderly patients as an alternative to conventional analgesics such as NSAIDs, which should be used rarely with extreme caution in this population.
- However, studies are required to investigate the long-term use of fixed-dose combinations.

## Figures and Tables

**Table 1:** Instruments for the assessment of pain and pain-related quality of life.

Questionnaire	Assessments	Scoring
Pain scales, for example, (i) numerical scales (NRS) [16] (ii) visual analogue scales (VAS) (iii) verbal rating scales (VRS) (iv) Wong-Baker FACES scales [17]	Assessment of pain intensity	Self-assessment and physician assessment 11-point or 100 mm scales 0 = no pain to 10 = worst imaginable pain

CHANGE PAIN scale [33] (i) 11-point NRS on the front (ii) 6 key parameters affecting quality of life on the back	Assessment of pain intensity Setting individual treatment goals for the patients	11-point scale with 0 = no pain to 10 = worst imaginable pain Need for improvement: not at all, a little, very much

SF-36-Health Survey [28]	Measures general health status including physical functioning, role-physical, bodily pain, general health, vitality, social functioning, role-emotional, and mental health An additional question determines the health status compared to the previous year	8 items are rated on a scale ranging from 0 to 100; higher values indicate a better outcome A frequently used shorter version is the SF-12 [34]

Short Form McGill Pain Questionnaire (SF-MPQ) [35]	Pain assessment with a short form of the McGill Pain Questionnaire It includes the Present Pain Intensity (PPI) index of the standard MPQ and a visual analogue scale (VAS).	15 items with sensory and affective subscores Pain over the last 7 days is rated on a 4-point categorical scale

Brief Pain Inventory (BPI) [36]	Measures pain (including presence of pain, localization, pain intensity, pain medication, and pain reduction during defined time frame) and impairments of daily life	Self-assessment Short version contains 9 questions scored on an 11-point scale ranging from 0 = no pain/no impairment to 10 = as bad as you can imagine

EuroQol (EQ-5D) [37]	Assessment of pain, quality of life, and daily functioning	Part 1 includes five dimensions: mobility, self-care, usual activities, pain/discomfort, and anxiety/depression For each dimension, three statements that best describe the patient's health status are selected Part 2: VAS health status rating scale from 0 (worst status) to 100 (best status).

Depression, Anxiety, and Stress Scale (DASS) [29]	Assessment of depression, anxiety, and stress	Self-assessment Three subscales with 14 items, rated on a four-point Likert scale (total scores from 0 to 42 on each subscale) Depression: scores above 20 indicate severe depression Anxiety: scores above 14 indicate severe anxiety Stress: scores above 25 indicate severe stress

Beck Depression Inventory (BDI) [30]	Sensory pain description and assessment of affective pain experience	Self-assessment 8 adjectives for the description of sensory components 4 adjectives for the description of affective components Scores from 0 to 63: 14–19 mild depression, 20–28 moderate depression, and 29–63 severe depression

Western Ontario and McMaster Universities (WOMAC) Osteoarthritis Index [31, 32]	Condition-specific Assessment of pain, stiffness, and difficulty in function	Patient self-assessment 24 questions in three domains: pain, disability, and joint stiffness Answers are scored on a 5-point Likert scale or 100 mm visual analogue scale with higher scores indicating greater difficulty

**Table 2:** The DN4 questionnaire (Douleur Neuropathique en 4 questions) to estimate the probability of neuropathic pain [19].

Interview of the patient		
*Question *1:		
Does the pain have one or more of the following characteristics?	Yes	No
Burning	□	□
Painful cold	□	□
Electric shocks	□	□
*Question *2:		
Is the pain associated with one or more of the following symptoms in the same area?	Yes	No
Tingling	□	□
Pins and needles	□	□
Numbness	□	□
Itching	□	□

Examination of the patient		
*Question *3:		
Is the pain located in an area where the physical examination may reveal one or more of the following characteristics?	Yes	No
Hypoesthesia to touch	□	□
Hypoesthesia to pinprick	□	□
*Question *4:		
In the painful area, can the pain be caused or increased by	Yes	No
Brushing?	□	□

**Table 3:** Fixed-dose tramadol/paracetamol for chronic pain: study designs.

Study	Type	Patients	Tramadol/paracetamol mean daily dose	Comparator mean daily dose
Musculoskeletal pain				

Mullican and Lacy, 2001 [85]	4-week double-blind, double-dummy, active-control, multicenter, randomized	462 Mean age 57.6 years Chronic nonmalignant low back pain, osteoarthritis pain, or both	131 mg/1133 mg	Codeine/paracetamol 105 mg/1054 mg
Serrie et al., 2011 [86]	Observational, prospective, open-label, in clinical practice 37.6% first-line treatment 62.4% following treatment failure of mainly paracetamol or analgesics containing dextropropoxyphene	5495 Mean age 53.2 ± 15.9 years Mainly musculoskeletal pain	139 mg/1203 mg Mean treatment duration 16.6 ± 9.8 days	None
Mejjad et al., 2011 [87]	Observational, prospective, open-label, in clinical practice 30% first-line treatment 70% after treatment failure/safety problems with at least one other analgesic	2,663 ≥65 years old; mean age 73.6 ± 6.6 years Primarily musculoskeletal pain	143 mg/1235 mg Mean treatment duration 23.2 ± 9.2 days	None

Osteoarthritis				

Silverfield et al., 2002 [96]	10-day multicenter, double-blind, placebo-controlled, randomized Add-on treatment	308 Mean age 60.1 ± 9.9 years Hip or knee flare pain	37.5 mg/325 mg 1-2 tablets qid	Placebo
Emkey et al., 2004 [97]	3-month multicenter, double-blind, randomized Add-on to celecoxib or rofecoxib	306 Mean age 61 ± 9 years Hip or knee pain	154 mg/1332 mg	Placebo
Park et al., 2012 [98]	Multicenter, randomized, comparative 8-week monotherapy after 4-week add-on to meloxicam or aceclofenac	97 Knee OA > 1 year	121 mg/1050 mg Mean age 60 ± 7.4 years	Meloxicam or aceclofenac Mean age 61.2 ± 7.5 years
Choi et al., 2007 [99]	2-week multicenter, double-blind, double-dummy add-on to NSAIDs Safety study Randomization to titration and nontitration group	250 Mean age 60.2 ± 7.8 years Knee OA on stable NSAID therapy	37.5 mg/325 mg tid Titration over 7 days for titration group	None

Low back pain				

Ruoff et al., 2003 [106]	3-month double-blind, multicenter, randomized, placebo-controlled	318 Mean age 53.9 years At least moderate chronic lower back pain	158 mg/1365 mg	Placebo
Peloso et al., 2004 [107]	3-month double-blind, multicenter, randomized, placebo-controlled	336 Mean age 57.5 ± 12.6 years At least moderate chronic lower back pain	158 mg/1369 mg	Placebo

Rheumatoid arthritis				

Lee et al., 2006 [108]	1-week, double-blind, randomized, placebo-controlled Add-on treatment	277 Inadequate pain control by conventional NSAIDs and DMARDs	112.5 mg/975 mg Mean age 51.6 ± 11.7 years	Placebo Mean age 52 ± 12 years

Painful diabetic neuropathy				

Freeman et al., 2007 [109]	66-day double-blind, multicenter, randomized, placebo-controlled	313 Mean age 55.7 ± 10.3 years 94% type 2 diabetics Neuropathy symptoms for a mean 3.7 ± 2.6 years	158 mg/1365 mg	Placebo
Ko et al., 2010 [110]	6-week, open-label, randomized	163 25–75 years Type 2 diabetes	Mean dose at final visit 158 mg/1371 mg Mean age 58.6 ± 7.5 years	Gabapentin Mean dose at final visit 1575 mg Mean age 57.1 ± 9.3 years

DMARD: disease-modifying antirheumatic drug; NSAID: nonsteroidal anti-inflammatory drug; OA: osteoarthritis.
